# Assessing the contributions of technical error and biological variability to error of measurement in reliability studies

**DOI:** 10.5114/biolsport.2026.160239

**Published:** 2026-03-16

**Authors:** Basilio Pueo, Will Hopkins

**Affiliations:** 1 Health, Physical Activity and Sports Technology (Health-Tech), Physical Education and Sports, Faculty of Education, University of Alicante, 03690 Alicante, Spain

**Keywords:** Variability, Spreadsheet, Bootstrap, Jump-height, Standardization

## Abstract

A neglected issue in reliability studies is the contribution of technical (device) error and biological variability to the standard (typical) error of measurement. These contributions can be estimated with mixed modeling when subjects are measured simultaneously with two devices on two occasions. The aim of this study was to develop and validate a spreadsheet-based method to separately estimate technical error and biological variability, and to compare its performance with a mixed model. Change scores between occasions and devices are combined to yield standard deviations (SDs) for technical errors and biological variability in raw, percent, and standardized units, with bootstrapped confidence limits. The analyses were reproduced in a computer program and evaluated by simulation for true values of means and SDs mimicking real data with sample sizes of up to 50. Bias (deviation from true values) and uncertainty (width of confidence intervals) in the means and SDs were assessed by standardization, and coverage of true values by confidence intervals was assessed as nominal, under or over. Empirical correction factors were derived to correct small-sample bias in the SDs and eliminate under-coverage. Spreadsheet estimates were then compared to those of a mixed model. Bias was trivial for all estimates. Uncertainty was similar for the spreadsheet and mixed model, although substantial even with the largest sample size. The spreadsheet generally outperformed the mixed model for coverage. Analysis of jump-height measurements of 31 young adults recorded simultaneously with a photoelectric device and a jump mat provided good evidence that the technical errors produced respectively trivial and substantial increases in the typical error. In conclusion, the spreadsheet is a trustworthy tool for evaluating device error.

## INTRODUCTION

Reliability refers to the reproducibility of outcomes from a measurement procedure when administered to the same individuals across multiple occasions. Better reliability represents more precise individual measurements and therefore better tracking of changes in research or practical situations. The most important measure of reliability is a standard deviation (SD) known as the typical or standard error of measurement, which is calculated from the changes within subjects between test and retest measurements [1]. A critical but often overlooked aspect of reliability is the fact that this SD consists of random biological variability, which each subject exhibits every time they are measured, combined with random technical error, which the measuring device adds to every measurement. Traditionally, these two sources of variability have been conflated into a single composite value representing the overall measurement error. Separating technical error from biological variability would allow assessment of device reliability independent of subject variability, which inevitably differs between types of subjects (young adults, athletes, the elderly, and so on).

The technical error can be estimated separately for some kinds of measurement, for example, the concentration of a biomarker in blood samples. Researchers can split the samples and analyze the splits by treating them like test and retest measurements. The resulting error of measurement is the technical error free of biological variability between samples and is often reported in the methods section as a coefficient of variation. This method is not appropriate for measurements of human performance or other behaviors, because it is not possible to split participants’ actions. Estimating the two components of measurement error is nevertheless possible by including simultaneous measurements with two devices during each test. Errors of measurement can be calculated across devices on each testing occasion. In this case, biological variability disappears from these errors of measurement, which now represent the combination of the technical errors of each device. Errors of measurement can also be calculated across testing occasions on each device, as in the usual reliability studies. Now the errors of measurement include the technical error and biological variability. From these estimates of standard error of measurement, it is possible to extract the estimates of the separate contributions of biological variability and technical errors.

One method of extracting the estimates is via a mixed model, as demonstrated by Pueo et al. [2]. Mixed models produce estimates of standard deviations for sources of variability along with their associated confidence intervals, representing sampling uncertainty. The use of mixed models can be problematic for sports practitioners lacking access to the specialized knowledge of a statistics consultant. Alternatively, simple algebraic equations can be used in a spreadsheet to estimate all the relevant standard deviations, but they cannot be used to estimate the uncertainties in the SDs.

The present study introduces the spreadsheet analysis. To deal with the uncertainties, we have used bootstrapping, by adapting a spreadsheet introducing the concept [3]. This approach is investigated through simulations in a computer program and compared with mixed modeling. Both approaches are then applied to real data from jump-height measurements. The aim is to provide a simple trustworthy method to estimate and evaluate device technical error and biological variability.

## MATERIALS AND METHODS

### Derivation of the spreadsheet method

**FIG. 1:**
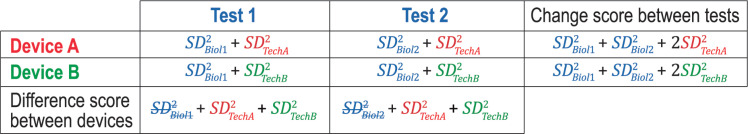
Variances of measurement error with two devices (A, B) on two testing occasions (1, 2) and variances of the change score between tests and of the difference score between devices. The variances consist of biological variability in the tests ($SD_{Biol1}^{2}$ and $SD_{Biol2}^{2}$) and technical error of two devices ($SD_{TechA}^{2}$ and $SD_{TechB}^{2}$).

The spreadsheet method is based on an algebraic partitioning of measurement error. The derivation requires a two-device, two-occasion study design, which yields variances from four key change scores. Figure 1 illustrates the composition of these variances from the constituent components of biological variability and technical error.

The technical errors and biological variability can be derived from the observed values of the variances of the change scores between the tests for each device and of the variances of the change scores between the devices for each test, as follows. The observed variances of the change scores between tests for Device A and Device B are given by:

$$SD_{A}^{2}=SD_{Biol1}^{2}+SD_{Biol2}^{2}+2SD_{TechA}^{2}$$ (1)

$$SD_{B}^{2}=SD_{Biol1}^{2}+SD_{Biol2}^{2}+2SD_{TechB}^{2}$$ (2)

The observed variances of the change scores between devices for Test 1 and Test 2 are given by:

$$SD_{1}^{2}=SD_{TechA}^{2}+SD_{TechB}^{2}$$ (3)

$$SD_{2}^{2}=SD_{TechA}^{2}+SD_{TechB}^{2}$$ (4)

Adding (3) to (4) and rearranging:

$$\frac{SD_{1}^{2}+SD_{2}^{2}}{2}=SD_{TechA}^{2}+SD_{TechB}^{2}$$ (5)

Subtracting (2) from (1) and rearranging,

$$\frac{SD_{A}^{2}-SD_{B}^{2}}{2}=SD_{TechA}^{2}-SD_{TechB}^{2}$$ (6)

Adding (5) to (6) and rearranging,

$$SD_{TechA}^{2}=\frac{\frac{SD_{A}^{2}-SD_{B}^{2}}{2}+\frac{SD_{1}^{2}+SD_{2}^{2}}{2}}{2}$$ (7)

$$SD_{TechB}^{2}=\frac{\frac{SD_{B}^{2}-SD_{A}^{2}}{2}+\frac{SD_{1}^{2}+SD_{2}^{2}}{2}}{2}$$ (8)

The variance representing biological variabilities on each Trial ($SD_{Biol1}^{2},SD_{Biol2}^{2}$) cannot be estimated separately, but their average ($SD_{Biol1}^{2},SD_{Biol2}^{2}$)/2) is given by combining and rearranging (1) with (7) or (2) with (8):

$$\frac{SD_{Biol1}^{2}+SD_{Biol2}^{2}}{2}=\frac{\frac{SD_{A}^{2}+SD_{B}^{2}}{2}-\frac{SD_{1}^{2}+SD_{2}^{2}}{2}}{2}$$ (9)

Assuming that the variance of the biological variability does not change between trials because no habituation or fatigue effects are present, the average variance is simple $SD_{Biol1}^{2}$. The spreadsheet produces this estimate of the biological variability.

### Subjects

To illustrate a practical application of this methodology, a study was conducted with thirty-one sports sciences students (22 males and 9 females). They were recreational athletes with varying abilities and training backgrounds. Subject characteristics (mean ± SD) for the males were age, 23.2 ± 1.7 years; height, 176 ± 7 cm; body mass, 75.0 ± 10.6 kg; those for the females were age, 21.8 ± 1.0 years; height, 165 ± 5 cm; body mass, 57.0 ± 5.3 kg. Exclusion criteria included lower-limb injuries and current medication use. Participants were instructed to abstain from alcohol and caffeinated beverages for 24 hours prior to testing. To control for circadian rhythm effects, each participant performed all jumps at the same time of day. This study was approved by the Human Research Ethics Committee of the University of Alicante (IRB No. UA-2019-02-25). All participants provided written informed consent before taking part in the study.

### Instrumentation

A photoelectric bar system (OptoJump, Microgate, Bolzano, Italy) and a jump mat (Axon Jump, Bioengineering Sports, Buenos Aires, Argentina) were used simultaneously to measure each jump test, referred to as Devices A and B, respectively. Both devices detect take-off and landing events, and they use the ballistic kinematic equation to estimate jump height [4]. The equation used is *h* = *t*^2^*g*/8, where *h* represents jump height (m), *t* is the flight time (s), and *g* is the acceleration due to gravity (9.81 m/s^2^).

The OptoJump system is an optical measurement tool designed to calculate temporal parameters during movement. It consists of two bars, each 1 m in length, equipped with infrared light-emitting diodes (LEDs) that transmit and receive signals. When an athlete jumps, any interruption in the communication between the bars is detected, allowing the system to measure flight time with a precision of 1 ms. The OptoJump system has been previously validated for jump height measurement [5, 6] and has been widely used in jump-performance research [7, 8]. Device B, the Axon Jump mat, consists of a 1.0 × 0.8 m platform that functions as a mechanical pressure switch and is connected to a laptop. The mat detects pressure changes when the athlete takes off and lands, enabling the calculation of flight time with a resolution of 1 ms. The Axon Jump system has been validated for jump height assessment [9] and has been utilized in various studies focused on jump-height performance [10, 11].

### Procedures

This observational study involved repeated measurements of maximum jump height during a single test session using two devices. Prior to jump execution, participants underwent a standardized 5-min warm-up on a cycle ergometer (Cardgirus Pro Medical, Alava, Spain) set at an 80-W power load and a cadence of 60 to 65 rpm. Following the warm-up, participants completed a series of familiarization jumps to practice proper technique and maintain balance during landing. Each participant then performed two countermovement jumps (CMJ), with a 1-min rest between repetitions. For correct execution, participants kept their hands on their hips, flexed their knees to a 90-degree angle, and executed the jump in a single continuous movement to achieve maximum height. Knee flexion was visually monitored in the sagittal plane, and any improperly performed jumps were repeated to ensure only successful trials were included in the analysis. The study was conducted at the Motion Analysis Laboratory (0001P1006) of the University of Alicante under controlled environmental conditions of approximately 22°C and 60% relative humidity.

### Statistical analyses

The statistical analysis proceeded in three stages: a simulation study to evaluate and adjust the spreadsheet method, a simulation study to evaluate and adjust mixed models, and the application of the spreadsheet and mixed modeling to the practical jump-height data.

The spreadsheet method was evaluated with simulations using SAS statistical software implemented on-line in SAS Studio (SAS 9.4; SAS Institute, Cary, NC). The SAS programs are extensively annotated and provided as supplementary material. We generated 4000 independent datasets mimicking reliability studies with a given sample size and pre-defined true population values for means and SDs. The context for the simulations was measurement of $\dot{\text{V}}$O_2max_, with population values of between- and within-athlete SDs (5.0% and 2.0% respectively) that could represent high-level athletes tested with devices that could have trivial and marginally moderate-large technical errors (0.3% and 3.0% respectively). For each simulated dataset, we applied the spreadsheet’s algebraic method. To quantify the uncertainty of the spreadsheet estimates, each dataset was bootstrapped with 1800 resamples (the maximum that could be included in the spreadsheet) to derive 90% confidence limits via the percentile method [3]. Bootstrapped variances were found to be biased in small samples, so the derived SDs were adjusted for this bias with an empirically derived factor. The SDs also showed the usual downward small-sample bias arising from taking the square root of variance, which was adjusted with a factor [12]. Width of bootstrapped confidence intervals for the means and SDs were found to be too narrow in small samples and were therefore adjusted with empirically derived factors to improve coverage. The spreadsheet incorporates these empirical adjustments, allowing for direct inspection of the formulas used to correct for bias and improve confidence interval coverage.

The performance of the spreadsheet was assessed by estimating bias (the difference between the true and simulated-mean values), uncertainty (the mean half-width of the 90% confidence interval), and coverage (the proportion of confidence intervals that included the true value, nominal being 90%). To assess the practical magnitude of the resulting errors, all outcomes were standardized. The spreadsheet and its simulation allow for standardization either with an external SD, or with either of two between-subject SDs provided by the data: the present SD (the observed SD in either trial, free of technical error) or the pure SD (the observed SD free of technical error and biological variability). For this study, standardization was performed using an external SD to permit meaningful evaluation of pure and present between-subject SDs. Sample values of the external SD were generated via a random variable with a chi-squared distribution. Bias arising from standardization was adjusted using the Becker factor [13] for means and an empirical modified Becker factor for SDs. Cohen’s modified and augmented thresholds were used to assess bias and uncertainty in means [14], and half these thresholds were used to assess SDs [15]. Thresholds for coverage were determined for 90% confidence intervals, which have a nominal expected error rate of 10% (10% of confidence intervals do not include the true value). Thresholds for count ratios (0.9, 0.7, 0.5, 0.3 and 0.1 for small, moderate, large, very large and extremely large reductions; the inverse of these for increases) [16] were applied to this nominal rate to determine the magnitudes for nominal coverage and for coverage substantially below and above nominal. Allowing for slight adjustment of some rounded values, nominal coverage is 89–91%; small, moderate, large, very large and extremely large over-coverage are 92–93%, 94–95%, 96–97%, 98–99%, and 100%; corresponding values for under-coverage are 88–86%, 85–80%, 79–67%, 66–1%, and 0%.

Simulated data were also used to evaluate two linear mixed models implemented with Proc Mixed in SAS. Simulations with poor or failed convergence of the mixed models were detected via zero standard error for the residuals and were excluded from further analysis. The proportion of such simulations was at most 3.5%, when the true technical errors were identical and sample size was the smallest (10). In the first mixed model, the technical error of the first device was estimated via the residual variance, while that of the second was estimated via the residual plus the extra variance of a dummy random-effect variable. This model was used when the true technical errors were different; it was also investigated with the residual estimating the larger technical error (and therefore estimating negative extra variance for the smaller technical error), but it performed less well (data not shown). In the second model, two residuals estimated the technical errors directly; this model was used when the true technical errors were identical, for which it performed better than the first model. In both models, a within-subject random effect estimated biological variability, and fixed effects for trial and device identities estimated mean changes between trials and mean differences between devices. The square roots of the variances and their confidence limits were adjusted for bias with the Gurland and Tripathi factor [12]. Confidence limits for differences and means of the SDs were estimated via parametric bootstrapping of the variances (their square roots being combined, after adjustment for bias), since the sampling distributions of such combinations of SDs are unknown. Parametric bootstrapping was also used for the confidence limits of the SDs themselves, since these were generally more accurate than those provided by the assumption of T distributions for the variances. The performance of the mixed models was assessed using standardization, as for the spreadsheet. The standard error for the standardized mean effects was augmented with the standard error arising from uncertainty in the standardizing SD, using a formula derived via first-order calculus to combine the standard errors. A similar formula was derived for the standardized variances. Bias arising from standardization was adjusted using the Becker factor for means and an empirical modified Becker factor for variances [13].

The jump-height data were analyzed with the spreadsheet and with the one-residual mixed model. Data were log-transformed before analysis, and standardization was performed with a value for an exterior SD [17]. Qualitative magnitudes and probabilistic inferences [14, 16, 18] replaced the analysis of bias, uncertainty and coverage.

## RESULTS

### Simulation performance

Table 1 shows the performance of the spreadsheet and mixed-model methods when simulating studies of sample size 30 with devices with unequal technical errors (true raw SDs of 0.30 and 3.0). Bias was trivial for all estimated parameters with both methods; the greatest bias occurred with the smaller of the technical errors, where the spreadsheet underestimated and the mixed model overestimated the true value. Uncertainties ranged from trivial to moderate and were similar for the spreadsheet and mixed model, although the spreadsheet uncertainty for the mean change between trials was a little wider. The spreadsheet method produced confidence interval coverage ranging from nominal (90–91%) to moderate over (94%), while coverage with the mixed model ranged from small under (86%) to very large over (98%). When the mixed model was run with the residual estimating the larger technical error, precision and coverage were a little worse for several SDs (data not shown).

**TABLE 1:** Performance of the spreadsheet (SS) and mixed model (MM) to estimate various means and standard deviations (SD) in 4000 simulations of reliability studies of two devices (A, B) on two occasions (Trial 1, Trial 2) with a sample size of 30, with chosen true raw values (shown in **bold**), and with standardized values estimated with an external standardizing SD drawn with a sample of size 30 from a population raw SD of 5.0 units. Bias, uncertainty and coverage were assessed with standardized values. Bias (the difference between true and estimated values) was trivial for all estimates. The raw values interpreted as percent units would be realistic in some settings.

Measure	Method	Raw values		Stdized values		Uncertainty^a^	Coverage^b^ (%)
	
		True	Est., ± CL	True	Est., ± CL		
Mean Trial 2 – Trial 1	SS	**2.0**	2.0, ± 1.1	**0.40**	0.40, ± 0.24	small	90, nominal
	MM		2.0, ± 0.9		0.40, ± 0.19	trivial	89, nominal

Mean Device B – Device A	SS	**3.0**	3.0, ± 0.7	**0.60**	0.60, ± 0.19	trivial	90, nominal
	MM		3.0, ± 0.6		0.60, ± 0.18	trivial	91, nominal

Pure between-subject SD	SS	**5.0**	5.0, ± 1.3	**1.00**	1.00, ± 0.36	moder.	92, small over
	MM		5.0, ± 1.2		1.01, ± 0.34	moder.	89, nominal

Within-subject SD	SS	**2.0**	2.0, ± 1.0	**0.40**	0.40, ± 0.22	small	94, moder. over
	MM		1.9, ± 0.6		0.39, ± 0.16	small	86, small under

Present between-subject SD	SS	5.4	5.4, ± 1.2	1.08	1.07, ± 0.36	moder.	92, small over
	MM		5.3, ± 1.1		1.08, ± 0.32	moder.	86, small under

Technical error A	SS	**0.3**	0.1, ± 1.6	**0.06**	0.02, ± 0.34	moder.	90, nominal
	MM		0.6, ± 2.1		0.11, ± 0.44	moder.	94, moder. over

Technical error B	SS	**3.0**	3.0, ± 0.6	**0.60**	0.60, ± 0.20	small	92, small over
	MM		2.9, ± 0.7		0.60, ± 0.19	small	92, small over

Technical error B – A	SS	2.7	2.9, ± 2.1	0.54	0.57, ± 0.45	moder.	90, nominal
	MM		2.4, ± 1.0		0.48, ± 0.26	small	90, nominal

Typical error A	SS	2.0	2.0, ± 0.5	0.40	0.40, ± 0.14	small	92, small over
	MM		2.0, ± 0.6		0.41, ± 0.16	small	92, small over

Typical error B	SS	3.6	3.6, ± 0.8	0.72	0.72, ± 0.24	small	92, small over
	MM		3.5, ± 0.6		0.71, ± 0.18	small	83, moder. under

Typical error B – A	SS	1.6	1.6, ± 0.8	0.32	0.31, ± 0.18	small	90, nominal
	MM		1.5, ± 0.6		0.30, ± 0.18	small	95, moder. over

Typical error A – within-subject SD	SS	0.0	0.0, ± 0.8	0.00	0.00, ± 0.16	small	94, moder. over
	MM		0.1, ± 1.3		0.03, ± 0.29	small	88, small under

Typical error B – within-subject SD	SS	1.6	1.6, ± 0.6	0.32	0.32, ± 0.15	small	94, moder. over
	MM		1.6, ± 0.7		0.32, ± 0.19	small	98, v.large over

Note: Stdized, standardized; Est., estimate provided by the method; CL, 90% confidence limits; moder., moderate; v.large, very large.

a Uncertainty refers to the standardized half-width of the confidence interval; magnitudes are defined by thresholds for means (< 0.20, trivial; 0.20–0.60, small) and for SDs (< 0.10, trivial; 0.10–0.30, small; 0.30–0.60, moderate).

b Coverage is the percentage of simulations where the confidence interval contained the true value; magnitudes for coverage are defined as: 80–85%, moderate under; 86–88%, small under; 89–91%, nominal; 92–93%, small over; 94–95%, moderate over; 96–97%, large over; 98–99%, very large over.

When the simulations were performed with identical devices (true raw technical error SD of 3.0 for both), bias was again trivial with either method (Table 2). Uncertainties were similar and small, with the exception of moderate for the within-subject SD. Coverage was under nominal only for the mixed-model’s mean typical error; coverage otherwise ranged from small to moderate over for the spreadsheet and from nominal to large over for the mixed model.

**TABLE 2:** Performance of the spreadsheet (SS) and mixed model (MM) as in Table 1, but with equal technical errors (3.0 raw units), to illustrate uncertainty and coverage when a study is performed with identical noisy devices and when relevant estimates of sums and differences of SDs are averaged. Bias was trivial for all estimates.

Measure	Method	Raw values		Stdized values		Uncertainty^a^	Coverage^b^ (%)
	
		True	Est., ± CL	True	Est., ± CL		
Technical error A	SS	**3.0**	3.0, ± 0.9	**0.60**	0.60, ± 0.24	small	93, small over
	MM		3.0, ± 0.9		0.61, ± 0.25	small	93, small over

Technical error B	SS	**3.0**	3.0, ± 0.9	**0.60**	0.60, ± 0.24	small	93, small over
	MM		3.0, ± 0.9		0.61, ± 0.25	small	93, small over

Technical error (A+B)/2	SS	3.0	3.0, ± 0.6	0.60	0.60, ± 0.19	small	93, small over
	MM		3.0, ± 0.6		0.61, ± 0.19	small	90, nominal

Within-subject SD	SS	**2.0**	2.0, ± 1.6	**0.40**	0.40, ± 0.35	moder.	94, moder. over
	MM		2.0, ± 1.5		0.40, ± 0.34	moder.	96, large over

Typical error A	SS	3.6	3.6, ± 0.8	0.72	0.72, ± 0.24	small	92, small over
	MM		3.6, ± 0.9		0.73, ± 0.22	small	89, nominal

Typical error B	SS	3.6	3.6, ± 0.8	0.72	0.72, ± 0.24	small	92, small over
	MM		3.6, ± 0.9		0.73, ± 0.22	small	89, nominal

Typical error (A+B)/2	SS	3.6	3.6, ± 0.6	0.72	0.72, ± 0.22	small	92, small over
	MM		3.6, ± 0.7		0.73, ± 0.18	small	87, small under

Typical error A – within-subject SD	SS	1.6	1.6, ± 1.4	0.32	0.32, ± 0.30	small	93, small over
	MM		1.6, ± 1.2		0.33, ± 0.29	small	96, large over

Typical error B – within-subject SD	SS	1.6	1.6, ± 1.4	0.32	0.32, ± 0.30	small	94, moder. over
	MM		1.7, ± 1.2		0.33, ± 0.29	small	95, moder. over

Typical error (A+B)/2 – within-subject SD	SS	1.6	1.6, ± 1.3	0.32	0.32, ± 0.27	small	93, small over
	MM		1.7, ± 1.1		0.33, ± 0.25	small	96, large over

Note: Stdized, standardized; Est., estimate provided by the method; CL, 90% confidence limits; moder., moderate.

a Uncertainty refers to the standardized half-width of the confidence interval; magnitudes are defined by thresholds for SDs (< 0.10, trivial; 0.10–0.30, small; 0.30–0.60, moderate).

b Coverage is the percentage of simulations where the 90% confidence interval contained the true value. Magnitudes for coverage are defined as: 86–88%, small under; 89–91%, nominal; 92–93%, small over; 94–95%, moderate over; 96–97%, large over.

Bias with sample sizes of 10 and 50 was trivial with all measures and models. Uncertainty and coverage with these sample sizes are summarized in Table 3. The models obviously performed better with the larger sample size, reaching trivial or small uncertainty and nominal coverage for means across all measures and models. Uncertainties for the spreadsheet were similar to those for the mixed model. There was more uncertainty in the SDs than in the means; with the larger sample size, the uncertainty in the SDs was still considerable (small or moderate), and there was still substantial (small) uncertainty with the change in the mean when technical errors were both 3.0. Coverage was better for the means than that for the SDs. Coverage for the spreadsheet was usually better than that for the mixed model, since the spreadsheet never showed under-coverage, and its over-coverage was often less than that of the mixed model.

**TABLE 3:** Performance of the spreadsheet (SS) and mixed model (MM) as in Tables 1 and 2, to illustrate uncertainty and coverage when studies are performed with sample sizes of 10 and 50, and with unequal or equal technical errors. Bias was trivial for all estimates.

Sample Size	TechA	TechB	Method	Means		SDs	
	
				Uncertainty	Coverage	Uncertainty^a^	Coverage^b^
10	0.3	3.0	SS	small	nom., small over	mod. – v.large	small over – v.large over
			MM	small	small under	mod. – large	mod. under – v.large over
	3.0	3.0	SS	small	nom., small over	mod. – v.large	mod. – v.large over
			MM	small	small under, nom.	mod. – v.large	small under – x.large over
50	0.3	3.0	SS	trivial	nom.	small – mod.	nom. – small over
			MM	triial	nom.	small – mod.	mod. under – v.large over
	3.0	3.0	SS	small, trivial	nom.	small	nom. – mod. over
			MM	small, trivial	nom.	small	small under – large over

Note: TechA, technical error of Device A; TechB, technical error of Device B; moder., moderate; v.large, very large; x.large, extremely large.

a Uncertainty refers to the standardized half-width of the 90% confidence interval. Magnitudes are defined by thresholds for means (< 0.20, trivial; 0.20–0.60, small) and for SDs (< 0.10, trivial; 0.10–0.30, small; 0.30–0.60, moderate; 0.60–1.2, large; 1.2–2.0, very large).

b Coverage is the percentage of simulations where the 90% confidence interval contained the true value. Magnitudes for coverage are defined as: 80–85%, moderate under; 86–88%, small under; 89–91%, nominal; 92–93%, small over; 94–95%, moderate over; 96–97%, large over; 98–99%, very large over; 100%, extremely large over.

### Jump-height reliability analysis

The analyses of the practical jump-height experiment are presented in Table 4. There was a small likely substantial increase in mean jump height on the second trial, but the difference in means between the devices was clearly trivial. The between- and within-subject standard deviations were clearly substantial, although their standardized uncertainties ranged from small (± 0.14, ± 0.13) to moderate (± 0.41 – ± 0.44). Spreadsheet and mixed-model estimates for these means and SDs were practically identical.

**TABLE 4:** Analyses with the spreadsheet (SS) and a mixed model (MM) of data from the reliability study of jump height measured with two devices (Device A, OptoJump; Device B, Axon jump mat) on two occasions (Trial 1, Trial 2). Standardization was performed with an external standard deviation (SD = 12.0%, sample size = 241). Qualitative magnitudes and probabilistic inferences were assessed with the standardized values.

Measure	Method	Percent values	Standardized values	Qualitative magnitude^a^	Probabilistic inference^b^
	
		Est., ± CL	Est., ± CL		
Mean Trial 2 – Trial 1	SS	3.8, ± 2.6	0.32, ± 0.22	small	↑**
	MM	3.8, ± 2.5	0.38, ± 0.21	small	↑**

Mean Device B – Device A	SS	−0.6, ± 1.1	−0.05, ± 0.10	trivial	↔ooo
	MM	−0.6, ± 1.0	−0.05, ± 0.09	trivial	↔oooo

Pure between-subject SD	SS	18.0, ± 4.8	1.44, ± 0.44	v.large	↑****
	MM	18.5, ± 4.5	1.50, ± 0.37	v.large	↑****

Within-subject SD	SS	5.4, ± 1.4	0.46, ± 0.14	moder.	↑****
	MM	5.6, ± 1.2	0.48, ± 0.11	moder.	↑****

Present between-subject SD	SS	18.9, ± 4.5	1.50, ± 0.44	v.large	↑****
	MM	19.5, ± 4.3	1.57, ± 0.35	v.large	↑****

Technical error A	SS	1.6, ± 2.8	0.13, ± 0.25	small	↑
	MM	0.0, ± 2.5	0.00, ± 0.22	trivial	↔

Technical error B	SS	4.5, ± 0.9	0.38, ± 0.10	moder.	↑****
	MM	4.7, ± 0.7	0.41, ± 0.07	moder.	↑****

Technical error B – A	SS	2.9, ± 3.5	0.25, ± 0.30	small	↑**
	MM	4.7, ± 1.2	0.40, ± 0.11	moder.	↑****

Typical error A	SS	5.6, ± 1.1	0.47, ± 0.13	moder.	↑****
	MM	5.6, ± 1.2	0.48, ± 0.11	moder.	↑****

Typical error B	SS	7.1, ± 1.5	0.60, ± 0.16	large	↑****
	MM	7.4, ± 1.1	0.63, ± 0.10	large	↑****

Typical error B – A	SS	1.4, ± 0.9	0.12, ± 0.08	small	↑*↔^o^
	MM	1.7, ± 0.6	0.15, ± 0.05	small	↑**

Typical error A – within-subject SD	SS	0.2, ± 0.6	0.01, ± 0.06	trivial	↔ooo
	MM	0.0, ± 0.3	0.00, ± 0.03	trivial	↔ooo

Typical error B – within-subject SD	SS	1.6, ± 0.5	0.14, ± 0.05	small	↑**
	MM	1.7, ± 0.6	0.15, ± 0.05	small	↑**

Note: Stdized, standardized; Est., estimate provided by the method; CL, 90% confidence limits; moder., moderate; v.large, very large.

a The qualitative magnitude is based on the standardized point estimate (Est.) and interpreted using the following thresholds. For means: < 0.20, trivial; 0.20–0.60, small. For SDs: < 0.10, trivial; 0.10–0.30, small; 0.30–0.60, moderate.

b ↑↓ Indicate substantial positive and negative effects, respectively; **↔** indicates trivial effects. Probabilities are shown for effects with adequate precision at the 90% level. Probabilities of substantial effects:

* , possibly;

** , likely;

*** , very likely;

**** , most likely. Probabilities of trivial effects:

o , possibly;

oo , likely;

ooo , very likely;

oooo , most likely.

Notable differences were observed in the estimation of the technical errors of the two devices. For the OptoJump, the mixed model’s estimate (0.0%) differed substantially from the spreadsheet’s exact sample value (1.6%), but both these estimates had inadequate precision, and their comparison (not performed) would also have been unclear. The estimates for the Axon were almost identical; they were moderate and clearly substantial, and the differences from the OptoJump were small with the spreadsheet (likely substantial) and moderate with the mixed model (most likely substantial).

The technical errors combined with within-subject variability to produce clearly substantial moderate (OptoJump) to large (Axon) typical errors, and the values for the spreadsheet and mixed model were practically identical. There was some evidence that the difference in the typical errors was small (possibly substantial for the spreadsheet, likely substantial for the mixed model). The contribution of the technical error to the typical error was clearly trivial for the OptoJump but small and likely substantial for the Axon, again with practically identical outcomes for the spreadsheet and mixed model.

## DISCUSSION

This study introduced and investigated a spreadsheet-based method for partitioning measurement error into its biological and technical components when analyzing data from a two-device, two-occasion reliability study. The components were expressed as SDs derived algebraically from variances of change and difference scores, and uncertainty on the SDs was determined with bootstrapping. Simulation was used to investigate the method’s performance, defined by bias and uncertainty in estimates of means and standard deviations, and by coverage of their confidence intervals. Performance was improved by implementing empirical corrections and was then compared with that of a linear mixed model. Our key finding is that the spreadsheet represents a trustworthy, accessible analytical tool, often superior to mixed modeling in relation to coverage with the small sample sizes common in sport and exercise science. This study also advances the conceptual approach to evaluating device error, in that the assessment of a device’s technical error should also be considered in the context of the subject’s biological variability: a given magnitude of technical error that might be substantial for a measure with low biological variability could be rendered negligible for a measure with high biological variability.

The bias in all means and SDs was trivial with both the spreadsheet and mixed-models, regardless of sample size and device errors (Tables 1–3). The qualitative magnitude of bias was derived by applying established magnitude thresholds to the standardized values, a process that makes our findings transferable but also contextdependent (for the simulations, measurement of $\dot{\text{V}}$O_2max_). The finding of trivial standardized bias indicates that our empirical corrections were successful in addressing the small-sample bias inherent in the spreadsheet estimates of SDs. However, bias may become substantial in a population with a smaller standardizing between-subject SD, particularly for the smaller of two technical errors estimated with the spreadsheet and mixed model.

The uncertainty of the estimates, represented by the confidencelimit half-width, was similar for both the spreadsheet and the mixed model across all conditions. As expected, uncertainty was smaller with the larger sample sizes, although it remained small to moderate for most SDs even with a sample size of 50 subjects, which is more than sport scientists would normally use in a study of reliability. The substantial uncertainty in the estimates is a critical consideration for researchers interpreting these values: most observed SDs in a real study with 50 subjects would have to be at least small to qualify for adequate precision, and they would have to be at least moderate to qualify for clearly substantial. As with bias, these qualitative labels are based on standardized values; the reported uncertainties should therefore be interpreted as specific to a population with a betweensubject SD similar to that of our simulated population.

Differences between the two methods emerged in the coverage of the confidence intervals. Being unaware of published thresholds for what constitutes poor coverage, we have applied thresholds previously suggested for count ratios to the nominal 10% error rate [16, 19]. The findings represent clear evidence for the spreadsheet’s superior performance in providing trustworthy estimates of uncertainty: the spreadsheet method consistently achieved either nominal (89–91%) or small to moderate over-coverage (92–95%) across all conditions and sample sizes; importantly, it never demonstrated under-coverage (Tables 1–3). In contrast, the mixed model frequently exhibited substantial under-coverage, particularly with smaller samples, falling as low as 86%. Over-coverage makes inferences more conservative, but under-coverage indicates that the confidence intervals are too narrow, leading to an over-confident and potentially misleading interpretation of the results. The superior performance of the spreadsheet is likely due in part to the empirical corrections we devised to improve coverage, and no such attempt was made with the mixed model. Confidence intervals in the spreadsheet were also derived with non-parametric bootstrapping, which does not rely on the assumption of normality for the sampling distributions of variances, an assumption that is violated in small samples. Calculation of confidence intervals with the mixed model requires this assumption.

The analysis of the practical jump-height data further highlighted a key advantage of the spreadsheet’s direct algebraic approach over the mixed model’s iterative algorithm. In the estimation of technical error for the OptoJump device, the mixed model produced a boundary estimate of 0.0% (Table 4), a value that seems implausible for this electronic device. The spreadsheet, in contrast, calculated the exact, non-zero sample value of 1.6%. Such boundary estimates in mixed models are a known issue, particularly with small samples or low variance components, where the estimation algorithm can fail to converge on a realistic value within the parameter space [20]. In these scenarios, the spreadsheet provides a more robust and practically meaningful sample estimate. The actual estimates for the technical error of the Optojump (small with the spreadsheet, trivial with the mixed model) were nevertheless unclear with both methods, which is consistent with the findings of the simulations with a sample size of 30. Nevertheless, the comparison of the two technical errors provided good evidence (spreadsheet) and strong evidence (mixed model) for greater technical error with the Axon jump mat. Furthermore, there was very good evidence that the Optojump contributed negligibly to the typical error, which defines the precision of measurements in practice, whereas there was good evidence that the jump mat contributed substantially to the typical error. Both devices work by estimating jump height from flight time (the time when the subject’s feet are not in contact with the jump mat), so the greater technical error in the jump mat must arise from mechanical activation in the timing circuit.

Our findings indicate that for a two-trial two-device design with small samples, the mixed model’s performance is suboptimal in relation to coverage, so an empirical correction similar to that for the spreadsheet could be investigated. The mixed model’s iterative algorithm would also benefit from the increased information provided by additional trials and/or simultaneous measurement by more than two identical devices, scenarios that are unwieldy or impractical for the spreadsheet. Future research could therefore investigate the performance of mixed models in this context. The mixed model could also be applied to samples bootstrapped directly from the original data, thereby avoiding the assumption of normality for estimation of confidence intervals.

An important caveat to the analyses presented here is the assumption that the technical error of measurement arising from the devices is due entirely to “reliability” error; that is, the error varies randomly from measurement to measurement within subjects, and there is no additional “validity” error, which would vary randomly between subjects. Validity error with measurement of jump height would arise, for example, from a difference in jumping style between subjects, such as consistent differences in the extent of knee flexion on landing. Jump height calculated from flight time by the Optojump and jump mat would be affected equally by such differences, so the validity SD representing such differences between subjects will have contributed to the between-subject SD in our analyses. Jump height estimated from video analysis of the excursion of a marker on the subject’s trunk would not be affected by knee flexion, and a comparison of this method against the Optojump or jump mat would require a statistical model that included SDs representing validity errors of each device. In a preliminary investigation of such a model, we have found that the SDs cannot be estimated algebraically with a spreadsheet. The mixed model can provide all the estimates, but adequate precision of the validity SDs requires larger sample sizes (several hundred, if the SDs are trivial or small), and of course, the validity SDs would be estimated only to the extent that the devices have functionally dissimilar methods of measurement.

## CONCLUSIONS

This study has introduced a spreadsheet that provides accessible trustworthy analysis of reliability data taken simultaneously with two devices. By estimating the contributions of biological variability and technical error to the error of measurement, our method provides a nuanced framework that allows researchers to make more informed judgments about a device’s utility for a specific population and measurement context. To facilitate its widespread adoption and to maximize the practical impact of this work, the validated spreadsheet has been made publicly available to the research community. The practical example highlights the relevance of this approach in contexts demanding precise measurement.

## Data Availability

The Excel spreadsheet tool (rely2devices.xlsx), which includes the raw jump-height data from the practical application presented in this study, is freely and publicly available for download from the University of Alicante institutional repository at the following permanent handle: http://hdl.handle.net/10045/161081 and in https://sportsci.org/resource/stats/rely2devices.xlsx. Users of the spreadsheet are kindly requested to cite the present journal article as the source of the method.
